# Lumbar Hernia Post Latissimus Dorsi Myocutaneous Flap Reconstruction for Breast

**DOI:** 10.1055/s-0045-1810434

**Published:** 2025-10-29

**Authors:** Soumyajyoti Panja, Tushar Dutta, Gautam Biswas

**Affiliations:** 1 Department of Surgical Oncology, Tata Medical Center, Kolkata, West Bengal, India; 2 Department of Reconstructive Plastic Surgery, Tata Medical Centre, Kolkata, West Bengal, India

**Keywords:** latissimus dorsi flap, lumbar hernia

## Abstract

We report a case of lumbar hernia in a patient with carcinoma in right breast who underwent mastectomy and reconstruction with a latissimus dorsi myocutaneous flap. Although rare, the diagnosis of lumbar hernia should be kept in mind in cases where latissimus dorsi myocutaneous flap is done to differentiate it from seroma (where aspiration is a form of management) and avoid potential complications such as bowel perforation. Treatment options are individualized, and can range from fascia closure, mesh reinforcement to minimally invasive laparoscopic and endoscopic techniques.

## Introduction

Few reported cases of lumbar hernia after breast reconstruction with latissimus dorsi myocutaneous flap exist in the literature. 1 The entity is so rare that Hafner et al 2 commented that a surgeon might encounter a lumbar hernia only once or twice in his or her lifetime. Although the latissimus dorsi flap remains a useful tool for autogenous breast reconstruction, it has its share of complications, which range from seroma formation, functional impairment, surgical site infection to marginal necrosis.

A case report of a patient with lumbar hernia will be discussed here: a rarely encountered complication post latissimus dorsi myocutaneous flap reconstruction.

## Clinical Scenario

**Fig. 1:**
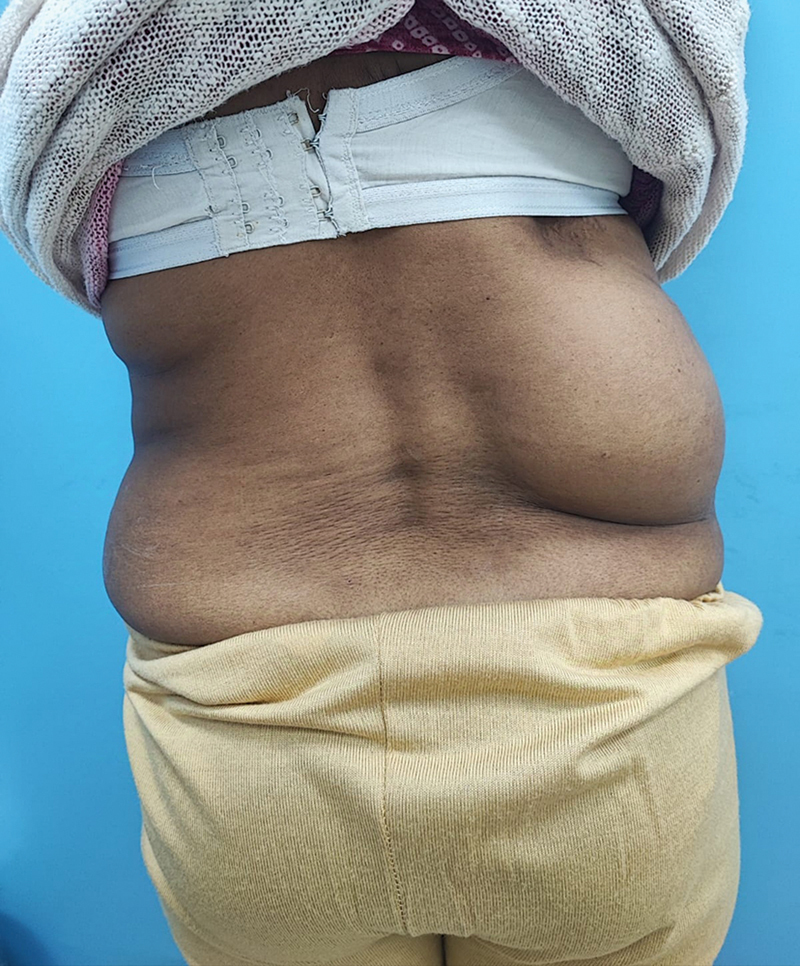
Right lumbar hernia in the donor site area of latissimus dorsi flap.

**Fig. 2:**
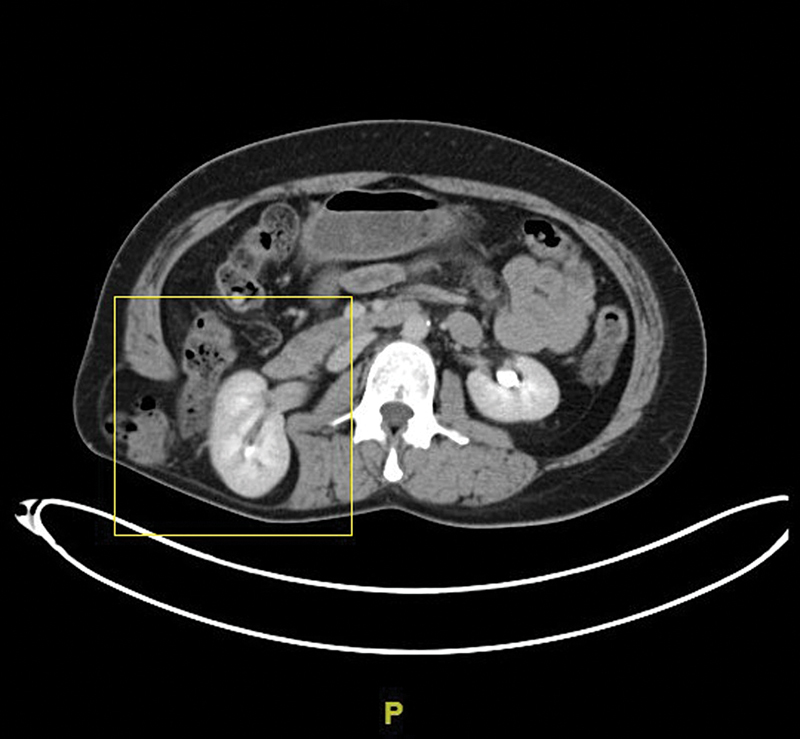
CT scan of abdomen demonstrating right lumbar hernia with colon and kidney as contents.

A 45-year-old woman from Assam who was diagnosed with carcinoma of right breast, underwent neoadjuvant chemotherapy followed by skin-sparing mastectomy and axillary lymph node dissection. Immediate breast reconstruction was performed with an extended latissimus dorsi flap. The surgery took place at a center different from the authors' institution. She developed postoperative seroma at the donor site, which later led to a surgical site infection, necessitating debridement and two sittings of secondary suturing. One year following the surgery, she noticed a bulge in the lumbar region around flap donor site. On clinical examination, there was a moderate size defect in the right lumbar area ( Fig. 1 ), where a swelling was apparent. The swelling was approximately 9 × 7 cm in dimension and firm in consistency. No tenderness was elicited on touch. It had a positive cough impulse and could be reduced without difficulty. A diagnosis of lumbar hernia was made. CT imaging suggested a right posterolateral abdominal wall defect of 7 × 6 cm in size containing cecum, ileocecal junction, ascending colon, and right kidney as contents ( Fig. 2 ).

## Surgical Technique

**Fig. 3:**
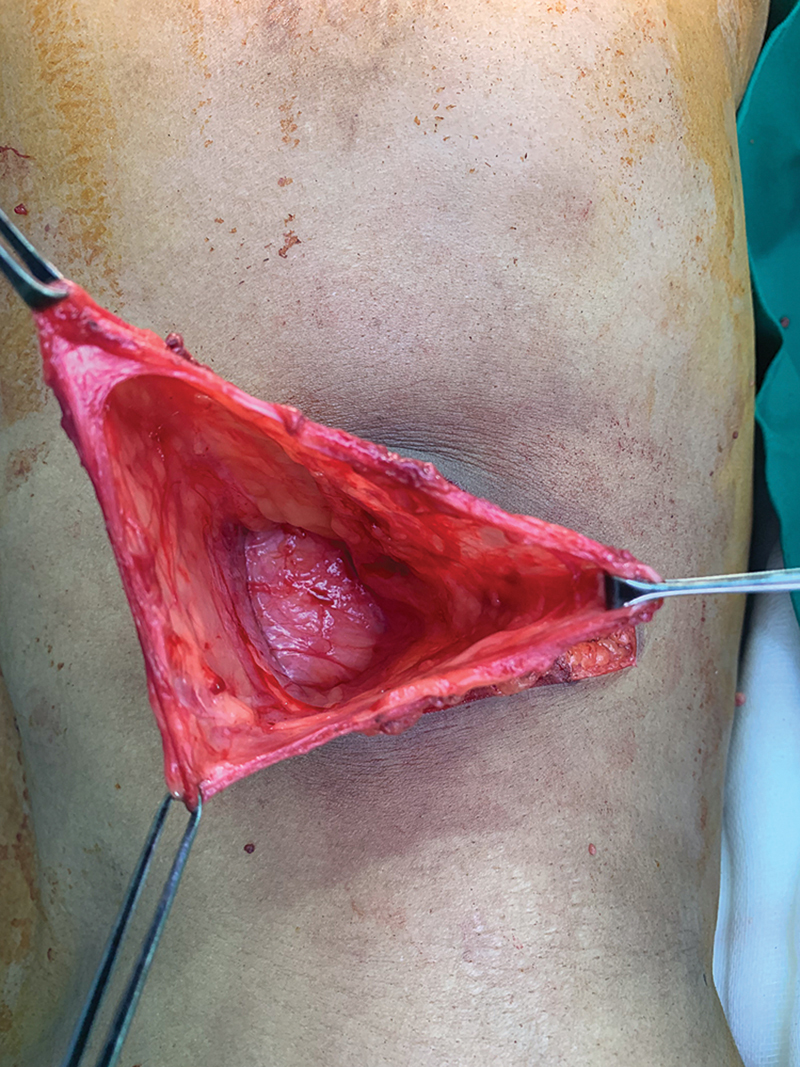
Opening of the hernia sac and reduction of its contents.

**Fig. 4:**
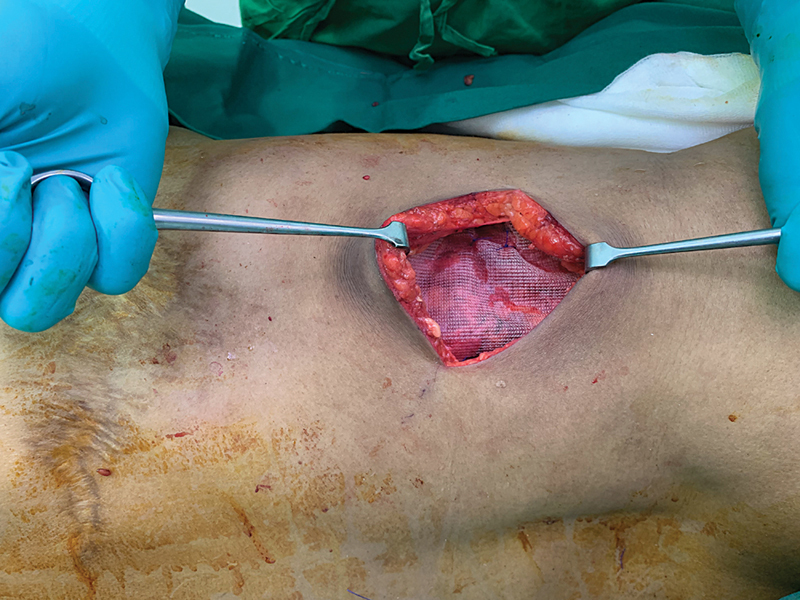
Mesh placement by onlay technique.

With the patient standing, a transverse incision was marked prior to surgery on the most prominent part of the bulge. Following incision dissection was performed to delineate the sac, which was noted to be arising from a defect in the superior lumbar triangle. The sac was opened, and the contents were reduced without difficulty ( Fig. 3 ). The sac was then excised and closed with 1–0 PDS running sutures. A Prolene mesh of size 15 × 15 cm was placed over the defect as an onlay, leaving at least 5 cm mesh all around ( Fig. 4 ). After securing hemostasis, a subcutaneous drain was placed, and wound was closed in layers.

## Follow-up

**Fig. 5:**
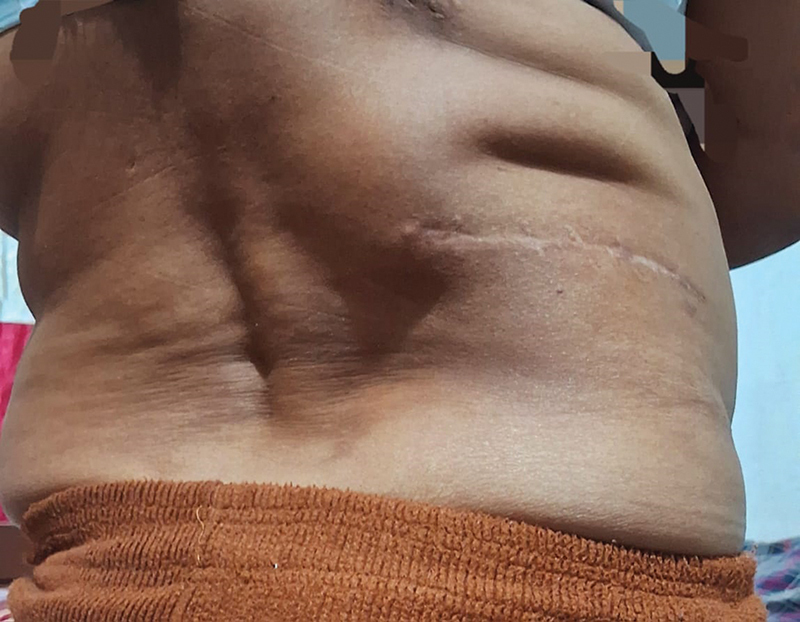
Postoperative scar examination during follow-up.

The postoperative course was uneventful; the patient was discharged on the second postoperative day. One year following the surgery, she is doing well with no evidence of weakness or any bulge at the operated site ( Fig. 5 ).

## Discussion

The latissimus dorsi originates from the spinous processes of thoracic 7th to 12th vertebrae, iliac crest, thoracolumbar fascia, and the inferior three ribs. The fibers converge into a tendon that attaches to the intertubercular sulcus of the humerus. It aids in extension, adduction, and medial rotation of the upper limb at the shoulder. It is innervated by the thoracodorsal nerve. 3

Lumbar hernias occur at two areas of the posterolateral abdominal wall: the superior and inferior lumbar triangle. 2 The superior lumbar triangle of Grynfeltt is a more frequent site. 2 It is an inverted triangle with its base formed by the 12th rib and the lower edge of the serratus muscle. The anterior border is formed by the internal oblique muscle while the posterior border is marked by the sacrospinalis muscle. The transversalis fascia forms the floor while the external oblique and latissimus dorsi muscle complete the roof of the triangle. The other less frequent area is the inferior lumbar triangle of Petit, which is bordered by the crest of the iliac bone at the base, the latissimus dorsi muscle medially, the external oblique muscle laterally, with its the floor consisting of the internal oblique muscle. 2 Etiological factors for lumbar hernia include congenital defects and trauma to the lumbar region. Idiopathic spontaneous herniation is also documented in the literature. 4

Additionally, incisional hernias can occur following procedures such as iliac crest bone harvest and renal surgeries. 4

In 1985, Moon and Dowden first mentioned the occurrence of lumbar hernia following latissimus dorsi flap reconstruction of breast. They cited chronic bronchitis and heavy lifting as possible risk factors. 1

Fraser et al opined that persistent pain and swelling in the lumbar region following latissimus dorsi harvest should raise suspicion of a lumbar hernia. 5 Differential diagnoses apart from seroma are tumors (like lipoma, fibroma, sarcoma etc.), hematoma, abscess, and renal hydrocele. Abdominal wall denervation atrophy may mimic lumbar hernia. However, it presents as an abdominal bulge instead of a true fascial defect.

Mickel et al described a right lumbar hernia after a delayed bilateral breast reconstruction. 6 The authors emphasized differential diagnosis with lumbar seroma and the importance of imaging of the lumbar region before proceeding to needle aspiration. They further stated that during flap harvest, dissection of the latissimus dorsi muscle should be done along its deep plane and one should transect the aponeurosis where it is attached to the deep fascia. This avoids compromising the deep fascia and prevents potential lumbar hernia formation.

For treatment of small hernias, mobilization of local tissue is sufficient to obtain adequate closure. For larger defects, additional reinforcement is achieved with a prosthetic mesh. 7

Minimally invasive techniques such as endoscopic, laparoscopic, and even retroperitoneoscopic approach to lumbar hernia repair have been described in the literature. 8 The positive aspects of these techniques are less pain, small incision, and a shorter hospital stay. Obregón et al first described the use of laparoscopy to treat a non-complicated superior lumbar hernia resulting from latissimus dorsi harvest for breast reconstruction. 9 The procedure has advantages like optimal exposure and characterization of the hernia defect as well as identification of its contents. It also permits an underlay repair and mesh fixation that can extend beyond the limits of the defect.

## Conclusion

Although a rare clinical entity, surgeons must be aware that lumbar hernia can occur following latissimus dorsi flap harvest. Differentiating it from seroma can help avert potential complications such as bowel perforation. The options for hernia repair vary from case to case and can range from fascial closure, mesh reinforcement to minimally invasive techniques such as laparoscopic and endoscopic repair.
